# Revisiting echocardiographic features of prosthetic heart valves: the necessity of correct differentiation of mono-leaflet vs. bileaflet mechanical heart valves in a case report

**DOI:** 10.1186/s13019-024-02633-x

**Published:** 2024-04-04

**Authors:** Farnoosh Larti, Babak Geraiely, Samaneh Hasanpour Asli, Arman Soltani Moghadam

**Affiliations:** Department of Cardiology, Imam Khomeini Hospital Complex, Tehran University of Medical Sciences, Keshavarz Boulevard, Tehran, Iran

**Keywords:** Mechanical heart valves, Echocardiography, Mono-leaflet, Bileaflet, Thrombosis, Cinefluoroscopy, Prosthetic heart valves

## Abstract

**Background:**

Mechanical heart valve replacement is a standard treatment for severe valvular disorders. The use of mono-leaflet valves has decreased recently. Recognizing the echocardiographic features of mono-leaflet and bileaflet valves is crucial for accurate complication diagnosis and proper management.

**Case Presentation:**

A 65-year-old female with mono-leaflet mitral and bileaflet tricuspid valves underwent an echocardiographic assessment. This simple educational case provides a unique opportunity to compare the echocardiographic features of these valves within a single patient.

**Conclusion:**

There is a crucial need for clinicians, particularly those in training, to differentiate between mono-leaflet and bileaflet mechanical heart valves adeptly. With mono-leaflet valves decreasing in prevalence, proficiency in recognizing the echocardiographic nuances of each type is imperative. Failure to do so may result in misdiagnoses and inappropriate management. This underscores the significance of continuous education and vigilance in echocardiographic assessments to ensure optimal patient care.

## Introduction

Mechanical heart valve replacement has long been the established treatment for patients with severe functional valve disorders. More than 200,000 valve replacement surgeries are being performed globally each year, and it has been estimated that this rate could boost up to 850,000 annually by 2050 [1]. The Starr–Edwards caged-ball model was the first mechanical heart valve utilized in 1960. However, its prevalence has waned over time due to an elevated susceptibility to thrombogenic complications [2]. Subsequently, in 1969, the innovation of mono-leaflet tilting valves marked a significant advancement. The Björk–Shiley tilting-disk valve, a prominent exemplar of this valve category, gained widespread utilization. In the modern era, St. Jude Medical bileaflet valve is the most abundant type of valve implanted, primarily used in 1977 [3]. Consequently, the pendulum has now completely swung towards utilizing bileaflet mechanical valves, necessitating familiarity with the distinctive echocardiographic and fluoroscopic views of mono-leaflet and bileaflet mechanical valves [4]. From an echocardiographic perspective, recognition of the characteristics and features of each valve is of particular importance in the detection of complications related to mechanical heart valves, with valve thrombosis being a particularly feared and consequential complication [2, 4].

As implantation of the mono-leaflet valves is constantly decreasing, younger cardiothoracic surgeons and cardiologists may need to familiarize themselves with the echocardiographic features of each valve. If not, they may mistake a “bileaflet valve with a fixed occluder” for a “mono-leaflet” valve, resulting in erroneous management. This educational case report presented the echocardiographic and correspondence fluoroscopic views of a patient with a simultaneous mono-leaflet mitral valve and a bileaflet tricuspid valve, providing the opportunity to compare the echocardiographic appearance in *one view*.

## Case presentation

A 65-year-old female patient was referred to our echocardiography lab for echocardiographic evaluation. She complains of mild dyspnea. The patient’s medical journey began 40 years ago when she was diagnosed with severe mitral valve stenosis and underwent a close mitral valve commissurotomy. Six years later, in 1987, she underwent a second cardiac operation (1st redo operation), during which a mono-leaflet valve (Björk–Shiley tilting-disk valve #27) was implanted. The patient was doing well till approximately two years ago, developing dyspnea and fatigue, leading to a further diagnosis of torrential tricuspid valve regurgitation. In response, she underwent a 2nd redo operation, and a mechanical bileaflet (St. Jude Medical bileaflet valve #31) was implanted. As the mono-leaflet mitral valve had a normal function in the pre-pump intra-operative transesophageal echocardiography (IO TEE), the surgeon performed MV-visiting and cleansing (removing the minimal pannus) and did not replace the mono-leaflet with a bileaflet one. The patient was highly concerned about redoing the intervention, so a bileaflet mechanical valve was chosen instead of bioprosthesis for treating tricuspid valve disease.

On this routine follow-up visit, about one year after her last surgery, the patient was referred for a comprehensive assessment of mechanical valves due to mild dyspnea. The echocardiography showed the normal mono-leaflet motion and hemodynamic assessment of the mitral valve (MG = 3.8mmHg, E velocity = 1.9 m/s, PHT = 64msec) and bileaflet tricuspid valve (MG = 5.3mmHg, E velocity = 2.2 m/s) with no evidence of paravalvular leakage in transthoracic echocardiography. In movie 1, the normal bileaflet motion of the TV occluders and the normal mono-leaflet motion of the MV occluder were presented. Movie 2 showed the cinefluoroscopic views, allowing a more detailed assessment of the mechanical valves. The relatively deep excursion of the single occluder into the left ventricle is the clue to diagnosing the mono leaflet mitral valve prosthesis. Before reaching this conclusion, one should be sure that there is no evidence of fixed occluder of a bileaflet mechanical prosthesis. As bileaflet mitral prosthesis is routinely implanted in an anti-anatomic position [5], the two occluders and their parallel motion are best apprehended in an apical two-chamber or off-axis short-axis view. Movie 3 and 4 represent the normal motion of the leaflet tricuspid valve and mono-leaflet mitral valve separately. For comparison, the echocardiography of another patient with a fixed lateral occlude was provided in Movie 5. Color doppler study of mitral and tricuspid prosthesis are shown in Movie 6 and 7.

## Discussion

Before echocardiography, reviewing the surgical report is necessary to prevent misdiagnosis. In case of unavailability of previous surgical reports, one should be able to differentiate the echocardiographic features of these two prosthesis valves. The simultaneous visualization of a bileaflet and a mono-leaflet valve in our patient offers a distinct educational advantage by providing a clear distinction between these valve types, thus mitigating potential diagnostic errors.

Prosthetic heart valves can be categorized into two main types: mechanical heart valves and bioprosthetic heart valves. Mechanical valves can be further classified into two major categories based on the design of the occluder: tilting disk (mono-leaflet) and bileaflet [6]. The leaflets’ opening and closing angles are particularly prominent when assessing valve function. Mono-leaflet valves are characterized by a single disc rotating around a central metal strut. The opening angle of the disk with the valve annulus spans from 60° to 80°, yielding two distinct orifices characterized by varying dimensions.

In contrast, bileaflet valves are crafted from two semilunar disks linked to a rigid valve ring through small pivots. The opening angle of the leaflets relative to the annulus plane varies between 75° and 90°. The open valve configuration encompasses three distinct orifices: a diminutive, elongated central orifice between the two open leaflets and two larger semicircular orifices positioned laterally [7]. To achieve maximum inflow, the surgeon implants the valve in an orientation resembling a native valve opening. Adherence to this issue is mostly important in mono leaflet mitral prosthesis to align the major orifice towards the aorta for greater stroke volume [8]. These valves are the most abundant type of mechanical valves being used to treat severe valvular heart disorders because of less thrombogenicity compared to mono-leaflet valves as well as better hemodynamic function, EOA, and less retrograde flow [3, 9–11]. Potential post-implantation complications, such as pannus formation and valve thrombosis, can be detected in both bileaflet and mono-leaflets. The interaction of the prosthesis with the host tissue results in the development of fibrous ingrowth or pannus. This occurrence has the potential to induce gradual obstruction. Additionally, valve thrombosis depends on the type of the heart valve and the patient’s associated risk factors, such as LV function and size or the presence of atrial fibrillation and inadequate anticoagulation [8].TTE is the first imaging modality to comprehensively assess prosthetic heart valves (PHVs). The cardiologist may encounter challenges in visualizing a leaflet. In such cases, complete assessment of patients with suspected PHV dysfunction often requires multimodality imaging, including transesophageal echocardiography, cine fluoroscopy, and cardiac CT.

TEE is a semi-invasive method capable of acquiring multiple high-quality images [12]. . Advanced echocardiographic techniques, including multiplane imaging and three-dimensional echocardiography, have been pivotal in identifying various mechanical valve types and associated dysfunction [13–16]. CT scans are also commonly used to determine prosthetic valve types and assess leaflet angles and excursions. Cinefluoroscopy is a frequently employed imaging modality for assessing prosthetic heart valve (PHV) function, detecting calcium deposition on leaflets, and visualizing valve motion. It is imperative to note that while cine fluoroscopy is valuable for these purposes and offers insights into the etiology of reduced disc mobility, its limitations include the inability to conduct hemodynamic assessments alongside a minor risk of exposure to radiation [4] Transesophageal Echocardiography (TEE) outperforms cinefluoroscopy by allowing comprehensive assessment of both motion and structure in prosthetic valves, particularly excelling in evaluating mitral and tricuspid valves. While cinefluoroscopy still plays a supplementary role in assessing disc mobility of mechanical aortic valves, TEE provides a superior diagnostic advantage. In challenging cases, multimodality imaging may be necessary for diagnostic precision and optimal care, particularly when a single modality is inconclusive for decision-making [17–20]. Moreover, recent developments in real-time 3D imaging, particularly from the transesophageal perspective, provide a significant added dimension for evaluating the function of prosthetic valves using the echocardiography [21].

In this lady who presented with mild dyspnea, we only proceeded with a cine fluoroscopy regarding the acceptable results of the transthoracic echocardiography and the presence of documented therapeutic INR. Visualizing valve leaflet mobility and measuring opening and closing angles makes identifying the valve type and potential complications possible [6].

## Data Availability

No datasets were generated or analysed during the current study.
